# *N*,*N*-Dimethyl­anilinium 2,4,6-trinitro­phenolate

**DOI:** 10.1107/S1600536808041743

**Published:** 2008-12-13

**Authors:** Nagarajan Vembu, Frank R. Fronczek

**Affiliations:** a Department of Chemistry, Urumu Dhanalakshmi College, Tiruchirappalli 620 019, India; b Department of Chemistry, Louisiana State University, Baton Rouge, LA 70803-1804, USA

## Abstract

In the title compound, C_8_H_12_N^+^·C_6_H_2_N_3_O_7_^−^, there are N—H⋯O and C—H⋯O inter­actions which generate *R*_2_^1^(5), *R*_2_^1^(6) and *R*_1_^2^(6) ring motifs. The supra­molecular aggregation is completed by the presence of edge-to-face and offset face-to-face π–π inter­actions with centroid–centroid distances of 3.673 and 3.697 Å, respectively.

## Related literature

For a detailed account of the design of organic polar crystals, see: Pecaut & Bagieu-Beucher (1993 ▶). For hydrogen bonding in nitro­phenol complexes, see: In *et al.* (1997 ▶); Zadrenko *et al.* (1997 ▶); Mizutani *et al.* (1998 ▶). For the supra­molecular architecture of mol­ecular complexes of trinitro­phenols, see: Botoshansky *et al.* (1994 ▶); Vembu *et al.* (2003 ▶). For details of the monoclinic polymorph of the title compound, see: Takayanagi *et al.* (1996 ▶). For hydrogen-bonding criteria, see: Desiraju & Steiner (1999 ▶); Desiraju (1989 ▶); Jeffrey (1997 ▶). For graph-set notation, see: Bernstein *et al.* (1995 ▶); Etter (1990 ▶).
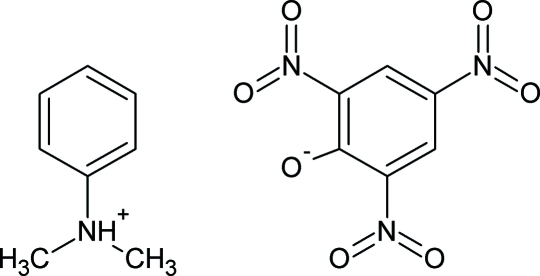


## Experimental

### 

#### Crystal data

C_8_H_12_N^+^·C_6_H_2_N_3_O_7_^−^
*M* *_r_* = 350.29
Orthorhombic, 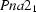

*a* = 15.9960 (10) Å
*b* = 9.1491 (6) Å
*c* = 10.3899 (9) Å
*V* = 1520.55 (19) Å^3^
*Z* = 4
Cu *K*α radiation
μ = 1.08 mm^−1^
*T* = 90.0 (5) K
0.26 × 0.24 × 0.08 mm

#### Data collection

Bruker Kappa APEXII CCD area-detector diffractometer
Absorption correction: multi-scan (*SADABS*; Sheldrick, 1996 ▶) *T*_min_ = 0.767, *T*_max_ = 0.919
16980 measured reflections
2823 independent reflections
2755 reflections with *I* > 2σ(*I*)
*R*_int_ = 0.033

#### Refinement

*R*[*F* ^2^ > 2σ(*F* ^2^)] = 0.023
*wR*(*F* ^2^) = 0.060
*S* = 1.03
2823 reflections
283 parameters
1 restraint
H atoms treated by a mixture of independent and constrained refinement
Δρ_max_ = 0.14 e Å^−3^
Δρ_min_ = −0.16 e Å^−3^
Absolute structure: Flack (1983 ▶), 1309 Friedel pairs
Flack parameter: 0.07 (12)

### 

Data collection: *APEX2* (Bruker, 2006 ▶); cell refinement: *APEX2* and *SAINT* (Bruker, 2006 ▶); data reduction: *SAINT*; program(s) used to solve structure: *SHELXS97* (Sheldrick, 2008 ▶); program(s) used to refine structure: *SHELXL97* (Sheldrick, 2008 ▶); molecular graphics: *PLATON* (Spek, 2003 ▶); software used to prepare material for publication: *SHELXL97*.

## Supplementary Material

Crystal structure: contains datablocks I, global. DOI: 10.1107/S1600536808041743/sj2565sup1.cif

Structure factors: contains datablocks I. DOI: 10.1107/S1600536808041743/sj2565Isup2.hkl

Additional supplementary materials:  crystallographic information; 3D view; checkCIF report

## Figures and Tables

**Table 1:** Hydrogen-bond geometry (Å, °)

*D*—H⋯*A*	*D*—H	H⋯*A*	*D*⋯*A*	*D*—H⋯*A*
N7—H7⋯O16	0.892 (17)	1.825 (18)	2.7128 (14)	172.8 (16)
N7—H7⋯O19	0.892 (17)	2.578 (16)	3.0517 (15)	114.0 (12)
C2—H2⋯O16	0.931 (18)	2.341 (17)	3.0464 (16)	132.4 (13)
C2—H2⋯O24	0.931 (18)	2.498 (17)	3.3444 (17)	151.4 (14)
C8—H8*A*⋯O19	1.001 (18)	2.411 (18)	3.1171 (18)	126.9 (13)
C9—H9*C*⋯O19	0.997 (18)	2.592 (17)	3.2311 (17)	121.9 (12)
C9—H9*B*⋯O21^i^	0.974 (19)	2.571 (19)	3.5074 (17)	161.3 (14)
C9—H9*B*⋯O25^ii^	0.974 (19)	2.476 (18)	3.0794 (18)	119.9 (13)
C4—H4⋯O21^iii^	0.924 (19)	2.466 (18)	3.1776 (16)	134.0 (14)
C14—H14⋯O19^iv^	0.941 (18)	2.564 (18)	3.4988 (16)	172.3 (14)
C9—H9*C*⋯O22^v^	0.997 (18)	2.502 (18)	3.3283 (16)	140.0 (13)

Symmetry codes: (i) 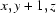; (ii) 
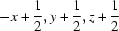
; (iii) 
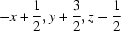
; (iv) 
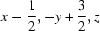
; (v) 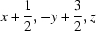.

**Fig. 1.**
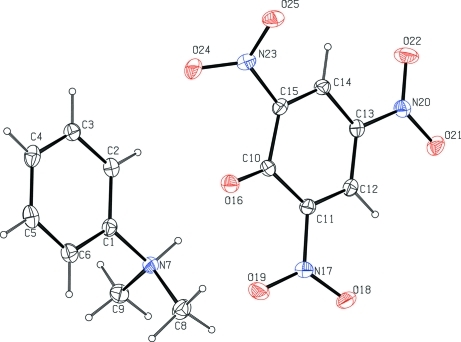
The asymmetric unit of (I) with the atoms labelled and displacement ellipsoids depicted at the 50% probability level for all non-H atoms. H-atoms are drawn as spheres of arbitrary radius.

**Fig. 2.**
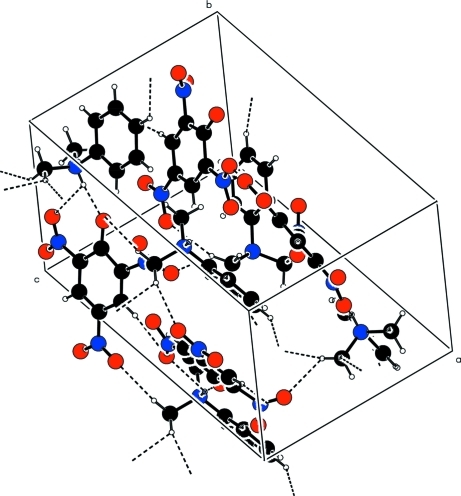
The molecular packing viewed down the b-axis. Dashed lines represent the N—H···O and C—H···O interactions within the lattice.

## supplementary crystallographic information

### Comment

The design of organic polar crystals for quadratic non-linear optical applications is supported by the observation that the organic molecules containing π-electron systems asymmetrized by electron donor and acceptor groups are highly polarizable entities in which problems of transparency and crystal growth may arise from their molecular crystal packing (Pecaut & Bagieu-Beucher, 1993). It is known that nitrophenols act not only as π-acceptors to form various π-stacking complexes with other aromatic molecules, but also as acidic ligands to form salts through specific electrostatic or H-bonding interactions (In *et al.*, 1997). The bonding of electron-donor acceptor complexes strongly depends on the nature of the partners. The linkage could involve not only electrostatic interactions, but also the formation of molecular complexes (Zadrenko *et al.*, 1997). It has been reported that proton transferred thermochromic complexes were formed between phenols and amines in apolar solvents at low temperature if an appropriate H-bonding network between the phenols and amines was present to stabilize it (Mizutani *et al.*, 1998). Pyridinium picrate has been reported in two crystalline phases and it appears in both phases as an internally linked H-bonded ion pair. These two phases are referred to as molecular crystals rather than salts based on their structural arrangements (Botoshansky *et al.*, 1994). A similar structural arrangement has also been reported for 4-dimethylaminopyridinium picrate (Vembu *et al.*, 2003). The monoclinic polymorph of the title compound (CSD Reference Code: REYDEE) has been reported previously (Takayanagi *et al.*, 1996). We have structurally elucidated the orthorhombic polymorph of the title compound as a forerunner to assessing its optical properties and report its structure here.

The asymmetric unit of (I) contains one *N*,*N*-Dimethylanilinium cation, and one 2,4,6-trinitrophenolate anion. (Fig.1). The crystal structure of (I) is stabilized by N—H···O and C—H···O interactions. The range of H···O distances (Table 1) found in (I) agrees with those found for N—H···O (Jeffrey, 1997) and C—H···O hydrogen bonds (Desiraju & Steiner, 1999). The N7—H7···O16 and N7—H7···O19 interactions form a pair of bifurcated donor bonds that link the *N*,*N*-dimethylanilinium cation and 2,4,6-trinitrophenolate anion and also form a motif of graph set *R*^2^_1_(6) (Bernstein *et al.*, 1995; Etter, 1990). Another pair of bifurcated donor bonds consists of the C2—H2···O16 and C2—H2···O24 interactions that also link the cation and the anion and form a *R*^2^_1_(6) motif. The C8—H8A···O19 and C9—H9C···O19 interactions constitute a pair of bifurcated bonds forming a *R*^1^_2_(6) motif that link the cation and the anion. The N7—H7···O19 and C8—H8A···O19 interactions constitute a pair of bifurcated acceptor bonds that form a *R*^1^_2_(5) motif. The above two motifs, *R*^1^_2_(6) and *R*^1^_2_(5), together form a *R*^1^_2_(5) motif by the interplay of the trifurcated acceptor bonds formed by N7—H7···O19, C9—H8A···O19 and C9—H9C···O19 interactions. There are five intermolecular C—H···O interactions (Table 1) that contribute to the supramolecular aggregation of the title compound. The intramolecular N—H···O interactions mentioned above also contribute to the formation of cooperative H-bonded network (Fig. 2). There is an offset π···π stacking interaction, Cg1···Cg2 (x, -1+y, z) at 3.697Å with α = 3.19, β = 24.88 and γ = 24.00 and the two perpendicular distances being  3.377 and 3.354Å. There is also an edge to face π···π stacking interaction, Cg1···Cg2 (1.5-x, -0.5+y, 0.5+z) at 3.673Å with α=13.75, β=25.68 and γ=12.78 and the two perpendicular distances being 3.582 and 3.310Å. Cg1 and Cg2 are the centroids of the C1···C6 and C10···.C15 rings.

The interplay of strong N—H···O and weak C—H···O, π···π interactions with different strengths, directional preferences and distance presents a complex mosaic of interactions. The three dimensional arrangement of the 2,4,6-trinitrophenolate and *N*,*N*-dimethylanilinium moieties in the unit cell, shows that the title compound is an internally linked hydrogen bonded ion pair and hence can be regarded as a molecular crystal rather than a salt.

### Experimental

2,4,6-Trinitrophenol (5.2 mmol) dissolved in aqueous ethanol (25 ml) was added dropwise to *N*,*N*-dimethylaniline (5.7 mmol) in aqueous ethanol (25 ml). The above solution was constantly stirred at room temperature for 2 hrs. The precipitated product was filtered and recrystallized from aqueous ethanol. Yield 75% (3.9 mmol).

### Refinement

All H-atoms were located in difference maps and their positions and isotropic displacement parameters freely refined. The 1309 Friedel pairs (96.2% coverage) were not merged, and the absolute structure was determined by refinement of the Flack (1983) parameter.

### Crystal data

**Table:**

C_8_H_12_N^+^·C_6_H_2_N_3_O_7_^−^	*D*_x_ = 1.530 Mg m^−^^3^
*M**_r_* = 350.29	Melting point: 401 K
Orthorhombic, *P**n**a*2_1_	Cu *K*α radiation, λ = 1.54178 Å
Hall symbol: P 2c -2n	Cell parameters from 9564 reflections
*a* = 15.996 (1) Å	θ = 5.5–70.2°
*b* = 9.1491 (6) Å	µ = 1.08 mm^−^^1^
*c* = 10.3899 (9) Å	*T* = 90 K
*V* = 1520.55 (19) Å^3^	Plate, yellow
*Z* = 4	0.26 × 0.24 × 0.08 mm
*F*(000) = 728	

### Data collection

**Table:**

Bruker Kappa APEXII CCD area-detector diffractometer	2823 independent reflections
Radiation source: fine-focus sealed tube	2755 reflections with *I* > 2σ(*I*)
graphite	*R*_int_ = 0.033
φ and ω scans	θ_max_ = 70.2°, θ_min_ = 5.6°
Absorption correction: multi-scan (*SADABS*; Sheldrick, 1996)	*h* = −19→19
*T*_min_ = 0.767, *T*_max_ = 0.919	*k* = −10→10
16980 measured reflections	*l* = −12→12

### Refinement

**Table:**

Refinement on *F*^2^	Secondary atom site location: difference Fourier map
Least-squares matrix: full	Hydrogen site location: inferred from neighbouring sites
*R*[*F*^2^ > 2σ(*F*^2^)] = 0.023	H atoms treated by a mixture of independent and constrained refinement
*wR*(*F*^2^) = 0.060	*w* = 1/[σ^2^(*F*_o_^2^) + (0.0386*P*)^2^ + 0.2146*P*] where *P* = (*F*_o_^2^ + 2*F*_c_^2^)/3
*S* = 1.03	(Δ/σ)_max_ < 0.001
2823 reflections	Δρ_max_ = 0.14 e Å^−^^3^
283 parameters	Δρ_min_ = −0.16 e Å^−^^3^
1 restraint	Absolute structure: Flack (1983), 1309 Friedel pairs
Primary atom site location: structure-invariant direct methods	Flack parameter: 0.07 (12)

### Special details

**Table:**

Geometry. All e.s.d.'s (except the e.s.d. in the dihedral angle between two l.s. planes) are estimated using the full covariance matrix. The cell e.s.d.'s are taken into account individually in the estimation of e.s.d.'s in distances, angles and torsion angles; correlations between e.s.d.'s in cell parameters are only used when they are defined by crystal symmetry. An approximate (isotropic) treatment of cell e.s.d.'s is used for estimating e.s.d.'s involving l.s. planes.
Refinement. Refinement of *F*^2^ against ALL reflections. The weighted *R*-factor *wR* and goodness of fit *S* are based on *F*^2^, conventional *R*-factors *R* are based on *F*, with *F* set to zero for negative *F*^2^. The threshold expression of *F*^2^ > σ(*F*^2^) is used only for calculating *R*-factors(gt) *etc*. and is not relevant to the choice of reflections for refinement. *R*-factors based on *F*^2^ are statistically about twice as large as those based on *F*, and *R*- factors based on ALL data will be even larger.

### Fractional atomic coordinates and isotropic or equivalent isotropic displacement parameters (Å^2^)

**Table:**

	*x*	*y*	*z*	*U*_iso_*/*U*_eq_	
C1	0.37023 (8)	1.31731 (14)	0.30638 (13)	0.0151 (3)	
C2	0.28455 (9)	1.29778 (15)	0.30264 (13)	0.0165 (3)	
C3	0.23581 (8)	1.40616 (15)	0.24585 (13)	0.0178 (3)	
C4	0.27257 (10)	1.53064 (15)	0.19438 (13)	0.0195 (3)	
C5	0.35871 (9)	1.54844 (15)	0.20120 (14)	0.0200 (3)	
C6	0.40878 (9)	1.44179 (15)	0.25752 (13)	0.0180 (3)	
N7	0.42143 (7)	1.20264 (12)	0.37028 (12)	0.0150 (2)	
C8	0.49639 (9)	1.15827 (16)	0.29319 (15)	0.0212 (3)	
C9	0.44558 (8)	1.25020 (15)	0.50295 (14)	0.0200 (3)	
C10	0.28722 (8)	0.85997 (14)	0.41862 (13)	0.0141 (3)	
C11	0.34012 (7)	0.75029 (14)	0.47878 (12)	0.0142 (2)	
C12	0.31105 (8)	0.62014 (14)	0.52863 (12)	0.0141 (3)	
C13	0.22695 (8)	0.58924 (13)	0.52313 (13)	0.0149 (3)	
C14	0.17059 (8)	0.68496 (14)	0.46412 (12)	0.0146 (3)	
C15	0.20036 (8)	0.81337 (13)	0.41512 (13)	0.0145 (3)	
O16	0.31146 (5)	0.97691 (10)	0.36915 (10)	0.0183 (2)	
N17	0.42940 (7)	0.77360 (12)	0.48867 (11)	0.0160 (2)	
O18	0.47489 (6)	0.66722 (10)	0.50973 (10)	0.0207 (2)	
O19	0.45735 (6)	0.89878 (10)	0.47807 (11)	0.0247 (2)	
N20	0.19719 (7)	0.45202 (12)	0.57365 (11)	0.0167 (2)	
O21	0.24919 (6)	0.36384 (10)	0.61486 (10)	0.0194 (2)	
O22	0.12141 (6)	0.42767 (12)	0.57227 (11)	0.0267 (2)	
N23	0.13932 (7)	0.90790 (12)	0.35088 (11)	0.0162 (2)	
O24	0.14260 (6)	1.04028 (10)	0.36928 (12)	0.0240 (2)	
O25	0.08701 (6)	0.84815 (11)	0.28202 (9)	0.0207 (2)	
H2	0.2611 (10)	1.2148 (19)	0.3398 (17)	0.017 (4)*	
H3	0.1778 (12)	1.3963 (19)	0.2403 (18)	0.023 (4)*	
H4	0.2380 (11)	1.600 (2)	0.1572 (16)	0.016 (4)*	
H5	0.3856 (11)	1.633 (2)	0.1698 (17)	0.023 (4)*	
H6	0.4678 (11)	1.4531 (17)	0.2608 (17)	0.017 (4)*	
H7	0.3883 (10)	1.1243 (18)	0.3740 (17)	0.018 (4)*	
H8A	0.5236 (11)	1.0734 (18)	0.3373 (18)	0.025 (4)*	
H8B	0.5363 (12)	1.2445 (19)	0.2860 (19)	0.028 (5)*	
H8C	0.4773 (12)	1.125 (2)	0.208 (2)	0.036 (5)*	
H9A	0.4780 (10)	1.3372 (18)	0.4959 (17)	0.017 (4)*	
H9B	0.3966 (11)	1.2756 (18)	0.5538 (18)	0.022 (4)*	
H9C	0.4760 (11)	1.1692 (19)	0.5467 (17)	0.020 (4)*	
H12	0.3479 (11)	0.5528 (18)	0.5656 (17)	0.017 (4)*	
H14	0.1134 (11)	0.6615 (17)	0.4590 (17)	0.017 (4)*	

### Atomic displacement parameters (Å^2^)

**Table:**

	*U*^11^	*U*^22^	*U*^33^	*U*^12^	*U*^13^	*U*^23^
C1	0.0177 (6)	0.0136 (6)	0.0140 (6)	0.0022 (5)	−0.0007 (5)	−0.0020 (5)
C2	0.0197 (6)	0.0135 (6)	0.0161 (6)	−0.0011 (5)	0.0012 (5)	−0.0010 (5)
C3	0.0172 (6)	0.0170 (6)	0.0193 (7)	0.0013 (5)	−0.0022 (5)	−0.0039 (5)
C4	0.0281 (7)	0.0146 (6)	0.0158 (6)	0.0041 (6)	−0.0028 (5)	−0.0002 (5)
C5	0.0286 (7)	0.0137 (7)	0.0175 (7)	−0.0029 (5)	0.0024 (6)	−0.0003 (5)
C6	0.0198 (7)	0.0166 (7)	0.0175 (7)	−0.0021 (5)	0.0024 (5)	−0.0017 (5)
N7	0.0142 (5)	0.0132 (5)	0.0174 (5)	−0.0006 (4)	0.0011 (4)	−0.0003 (5)
C8	0.0193 (6)	0.0192 (7)	0.0251 (7)	0.0028 (5)	0.0058 (6)	0.0008 (6)
C9	0.0214 (6)	0.0200 (7)	0.0187 (7)	0.0008 (6)	−0.0046 (6)	−0.0016 (6)
C10	0.0146 (6)	0.0140 (6)	0.0137 (6)	−0.0002 (5)	−0.0013 (5)	−0.0026 (5)
C11	0.0132 (6)	0.0156 (6)	0.0138 (6)	0.0004 (5)	0.0006 (5)	−0.0023 (5)
C12	0.0161 (6)	0.0136 (6)	0.0127 (6)	0.0030 (5)	−0.0003 (5)	−0.0009 (5)
C13	0.0170 (6)	0.0120 (6)	0.0155 (6)	0.0002 (5)	0.0001 (5)	0.0005 (5)
C14	0.0123 (6)	0.0162 (6)	0.0152 (6)	0.0002 (5)	0.0008 (5)	−0.0023 (5)
C15	0.0149 (6)	0.0140 (6)	0.0145 (6)	0.0031 (5)	−0.0007 (5)	0.0000 (5)
O16	0.0177 (4)	0.0152 (5)	0.0219 (5)	−0.0020 (3)	−0.0016 (4)	0.0040 (4)
N17	0.0142 (5)	0.0177 (5)	0.0162 (5)	−0.0005 (4)	−0.0008 (4)	0.0008 (5)
O18	0.0143 (4)	0.0205 (5)	0.0274 (5)	0.0040 (4)	−0.0012 (4)	0.0025 (4)
O19	0.0181 (5)	0.0180 (5)	0.0378 (6)	−0.0050 (4)	−0.0061 (4)	0.0062 (5)
N20	0.0166 (5)	0.0149 (6)	0.0186 (6)	−0.0005 (4)	−0.0009 (5)	0.0007 (4)
O21	0.0201 (4)	0.0144 (5)	0.0235 (5)	0.0035 (4)	−0.0005 (4)	0.0049 (4)
O22	0.0146 (5)	0.0267 (5)	0.0389 (6)	−0.0060 (4)	−0.0035 (4)	0.0098 (5)
N23	0.0135 (5)	0.0193 (6)	0.0156 (5)	0.0017 (4)	0.0009 (4)	0.0034 (5)
O24	0.0221 (5)	0.0140 (5)	0.0360 (6)	0.0036 (4)	−0.0007 (5)	0.0041 (4)
O25	0.0162 (4)	0.0271 (5)	0.0188 (5)	0.0020 (4)	−0.0047 (4)	0.0004 (4)

### Geometric parameters (Å, °)

**Table:**

C1—C2	1.3826 (19)	C9—H9C	0.997 (18)
C1—C6	1.3910 (19)	C10—O16	1.2488 (17)
C1—N7	1.4874 (16)	C10—C15	1.4537 (18)
C2—C3	1.3926 (19)	C10—C11	1.4538 (18)
C2—H2	0.931 (18)	C11—C12	1.3794 (19)
C3—C4	1.389 (2)	C11—N17	1.4475 (16)
C3—H3	0.934 (18)	C12—C13	1.3758 (18)
C4—C5	1.389 (2)	C12—H12	0.935 (18)
C4—H4	0.924 (19)	C13—C14	1.3985 (18)
C5—C6	1.391 (2)	C13—N20	1.4417 (17)
C5—H5	0.942 (19)	C14—C15	1.3661 (18)
C6—H6	0.951 (18)	C14—H14	0.941 (18)
N7—C9	1.4962 (18)	C15—N23	1.4652 (16)
N7—C8	1.4980 (18)	N17—O19	1.2344 (14)
N7—H7	0.892 (17)	N17—O18	1.2348 (14)
C8—H8A	1.001 (18)	N20—O22	1.2326 (15)
C8—H8B	1.017 (18)	N20—O21	1.2353 (15)
C8—H8C	0.99 (2)	N23—O24	1.2273 (15)
C9—H9A	0.953 (17)	N23—O25	1.2291 (15)
C9—H9B	0.974 (19)		
			
C2—C1—C6	122.34 (12)	H9A—C9—H9B	106.3 (14)
C2—C1—N7	117.85 (11)	N7—C9—H9C	109.3 (10)
C6—C1—N7	119.76 (11)	H9A—C9—H9C	113.0 (14)
C1—C2—C3	118.35 (12)	H9B—C9—H9C	108.9 (14)
C1—C2—H2	119.5 (10)	O16—C10—C15	122.53 (12)
C3—C2—H2	122.1 (10)	O16—C10—C11	125.98 (11)
C4—C3—C2	120.66 (13)	C15—C10—C11	111.39 (11)
C4—C3—H3	118.4 (11)	C12—C11—N17	115.67 (11)
C2—C3—H3	120.9 (11)	C12—C11—C10	124.13 (11)
C3—C4—C5	119.77 (13)	N17—C11—C10	120.21 (11)
C3—C4—H4	117.9 (11)	C13—C12—C11	119.44 (12)
C5—C4—H4	122.3 (11)	C13—C12—H12	119.8 (10)
C4—C5—C6	120.67 (13)	C11—C12—H12	120.7 (10)
C4—C5—H5	122.1 (11)	C12—C13—C14	121.33 (12)
C6—C5—H5	117.2 (11)	C12—C13—N20	119.13 (11)
C1—C6—C5	118.20 (13)	C14—C13—N20	119.47 (11)
C1—C6—H6	121.1 (10)	C15—C14—C13	118.50 (11)
C5—C6—H6	120.7 (10)	C15—C14—H14	121.0 (10)
C1—N7—C9	110.38 (10)	C13—C14—H14	120.5 (10)
C1—N7—C8	113.16 (11)	C14—C15—C10	125.18 (12)
C9—N7—C8	111.39 (11)	C14—C15—N23	116.42 (11)
C1—N7—H7	105.0 (10)	C10—C15—N23	118.37 (11)
C9—N7—H7	110.3 (12)	O19—N17—O18	122.24 (10)
C8—N7—H7	106.3 (11)	O19—N17—C11	119.19 (10)
N7—C8—H8A	108.2 (10)	O18—N17—C11	118.55 (10)
N7—C8—H8B	109.3 (11)	O22—N20—O21	123.25 (11)
H8A—C8—H8B	111.3 (14)	O22—N20—C13	118.55 (11)
N7—C8—H8C	108.4 (12)	O21—N20—C13	118.20 (10)
H8A—C8—H8C	108.1 (15)	O24—N23—O25	123.96 (11)
H8B—C8—H8C	111.3 (16)	O24—N23—C15	118.88 (11)
N7—C9—H9A	108.2 (11)	O25—N23—C15	117.15 (11)
N7—C9—H9B	111.2 (11)		
			
C6—C1—C2—C3	1.1 (2)	C12—C13—C14—C15	−2.10 (19)
N7—C1—C2—C3	178.45 (12)	N20—C13—C14—C15	−178.99 (12)
C1—C2—C3—C4	0.0 (2)	C13—C14—C15—C10	0.2 (2)
C2—C3—C4—C5	−0.9 (2)	C13—C14—C15—N23	178.23 (11)
C3—C4—C5—C6	0.9 (2)	O16—C10—C15—C14	177.99 (13)
C2—C1—C6—C5	−1.1 (2)	C11—C10—C15—C14	1.40 (19)
N7—C1—C6—C5	−178.45 (12)	O16—C10—C15—N23	0.00 (19)
C4—C5—C6—C1	0.1 (2)	C11—C10—C15—N23	−176.58 (11)
C2—C1—N7—C9	−100.90 (13)	C12—C11—N17—O19	−161.31 (13)
C6—C1—N7—C9	76.55 (15)	C10—C11—N17—O19	19.03 (18)
C2—C1—N7—C8	133.51 (13)	C12—C11—N17—O18	17.45 (17)
C6—C1—N7—C8	−49.04 (16)	C10—C11—N17—O18	−162.20 (12)
O16—C10—C11—C12	−177.78 (13)	C12—C13—N20—O22	177.52 (12)
C15—C10—C11—C12	−1.34 (18)	C14—C13—N20—O22	−5.53 (19)
O16—C10—C11—N17	1.8 (2)	C12—C13—N20—O21	−3.32 (18)
C15—C10—C11—N17	178.28 (11)	C14—C13—N20—O21	173.63 (12)
N17—C11—C12—C13	−179.98 (11)	C14—C15—N23—O24	138.87 (13)
C10—C11—C12—C13	−0.3 (2)	C10—C15—N23—O24	−42.97 (17)
C11—C12—C13—C14	2.17 (19)	C14—C15—N23—O25	−40.50 (17)
C11—C12—C13—N20	179.06 (12)	C10—C15—N23—O25	137.66 (12)

### Hydrogen-bond geometry (Å, °)

**Table:**

*D*—H···*A*	*D*—H	H···*A*	*D*···*A*	*D*—H···*A*
N7—H7···O16	0.892 (17)	1.825 (18)	2.7128 (14)	172.8 (16)
N7—H7···O19	0.892 (17)	2.578 (16)	3.0517 (15)	114.0 (12)
C2—H2···O16	0.931 (18)	2.341 (17)	3.0464 (16)	132.4 (13)
C2—H2···O24	0.931 (18)	2.498 (17)	3.3444 (17)	151.4 (14)
C8—H8A···O19	1.001 (18)	2.411 (18)	3.1171 (18)	126.9 (13)
C9—H9C···O19	0.997 (18)	2.592 (17)	3.2311 (17)	121.9 (12)
C9—H9B···O21^i^	0.974 (19)	2.571 (19)	3.5074 (17)	161.3 (14)
C9—H9B···O25^ii^	0.974 (19)	2.476 (18)	3.0794 (18)	119.9 (13)
C4—H4···O21^iii^	0.924 (19)	2.466 (18)	3.1776 (16)	134.0 (14)
C14—H14···O19^iv^	0.941 (18)	2.564 (18)	3.4988 (16)	172.3 (14)
C9—H9C···O22^v^	0.997 (18)	2.502 (18)	3.3283 (16)	140.0 (13)

Symmetry codes: (i) *x*, *y*+1, *z*; (ii) −*x*+1/2, *y*+1/2, *z*+1/2; (iii) −*x*+1/2, *y*+3/2, *z*−1/2; (iv) *x*−1/2, −*y*+3/2, *z*; (v) *x*+1/2, −*y*+3/2, *z*.
